# Quality of life profiles and their associations with depressive symptoms and cognitive impairment of community-dwelling older adults in Hong Kong

**DOI:** 10.3389/fpubh.2023.1165934

**Published:** 2023-05-18

**Authors:** Guozhi Luo, Weiping Li, Donghai Wu, Xinyue Wei, Yanpeng Zang, Jing-Dong Liu

**Affiliations:** ^1^Department of Geriatric Psychiatry, Shenzhen Kangning Hospital, Shenzhen, China; ^2^Weighting and Combat Sports Administrative Center of Guangdong, Guangzhou, China; ^3^Department of Physical Education, Sun Yat-Sen University, Guangzhou, China; ^4^Department of Physiotherapy, School of Nursing and Health Studies, Hong Kong Metropolitan University, Hong Kong, Hong Kong SAR, China

**Keywords:** quality of life, depression, cognitive impairment, older adults, latent profile analysis

## Abstract

**Background:**

This study aimed to (1) explore the quality of life (QoL) profiles of older adults in Hong Kong and (2) examine their association with predictors (age, sex, body mass index, and depressive symptoms) and distal outcome (cognitive impairment) using a person-centered approach.

**Methods:**

A total number of 328 community-dwelling older adults in Hong Kong were invited to participate in this study. Data from 259 older adults were identified as valid for the primary analysis. Latent profile analysis was used to explore QoL profiles. Multinomial logistic regression using the R3STEP function in Mplus was used to explore the predictive role of age, sex, body mass index, and depressive symptoms in profile membership. The Bolck-Croon-Hagenaars approach was used to examine how the distal outcome of cognitive impairment differs as a function of QoL profiles.

**Results:**

Three QoL profiles emerged from the latent profile analysis (Low, Moderate and High QoL). It was found that depression, but not age, sex, or body mass index, significantly predicted QoL profile membership. The results of the Bolck-Croon-Hagenaars analysis revealed no significant differences in cognitive impairment across the three QoL profiles.

**Conclusion:**

This is the first study that examined the relationship between QoL, depressive symptoms and cognitive impairment of older adults using a person-centered approach. The findings provide additional information for the evidence obtained from variable-centered approach on the associations among variables abovementioned. Our additional focus on the antecedents of emergent QoL profiles also provide practical knowledge regarding timely treatment for or prevention of depressive symptoms, which we submit will be crucial for enhancing the QoL of older adults.

## 1. Introduction

Life expectancy in Hong Kong (HK) has steadily increased over the past few decades. According to the Hong Kong Census and Statistics Department (1), the older adult population, those individuals who are at least 65 years of age, will reach 2.58 million (35%) by 2069, compared to 1.32 million (17.5%) in 2019. Aging is usually accompanied by a decline in physical and cognitive functioning (2) and therefore, the dramatic increase in life expectancy and, consequently, the size of the aging population is likely to create economic and social challenges for the government. In the 2015 financial year, the HK government spent HK$55.3 billion on the older adults, and this figure is expected to increase to HK$142.9 billion by 2064. In addition, a high suicide rate has been observed in older adults in HK, with an average rate of 26.8% for the period 1981 to 2015. In 2014, older adults comprised 15% of the HK population but accounted for 29.3% of all deaths due to suicide. This is a much larger proportion than that of other age groups (3). Both the physical and mental components of quality of life (QoL) have been identified as main factors that precipitate suicide in older adults (3). Previous research has also revealed that QoL can be used to predict cardiovascular disease, future hospitalization, and all-cause mortality (4–6). In light of these alarming statistics and significant consequences, it is submitted that the HK government should pay attention to the QoL of the older adult population, as it is not only important for the wellbeing of older adults in general, but also the happiness of thousands of families and the development of society (7).

Researchers have extensively explored and examined the concept and constructs of QoL. It is widely accepted that QoL is a multidimensional concept, although consensus on the specific components of QoL has not yet been achieved (7–11). According to the World Health Organization (WHO) definition, QoL refers to “an individual’s perception of their position in life in the context of the culture and value systems in which they live and in relation to their goals, expectations, standards, and concerns” (12). This definition emphasizes that QoL is a subjective view of one’s life and is influenced by various factors (13, 14). Previous research has thoroughly investigated the factors that may affect QoL in older adults. Depressive symptoms have consistently been found to be negatively associated with QoL in older adults (15–19). A meta-analysis review summarizing the results of 84 studies revealed that older adults with depression had poorer QoL than older adults without depression, and that an increase in depression severity was associated with a poorer QoL (9). More importantly, these associations were found to be stable over time and independent of how QoL was assessed. However, previous findings on the relationships between QoL and age, body mass index (BMI), and sex among older adults were mixed. For example, previous research in the United Kingdom (UK) has suggested that the QoL of older adults decreased significantly over 4 years, with a more rapid decline rate in later life (20). In contrast, results from studies conducted on older persons with mild cognitive impairment in China revealed very little change in QoL over 4 years (21), which suggests that a decline in QoL with age is not inevitable. Similar results were also reported in another longitudinal study, in which no substantial relationship between age and average change in QoL scores among adults aged ≥70 years was observed (22). This is consistent with Netuveli and Blane’s argument that an extended period of good QoL is possible since aging does not automatically negatively influence QoL (23). Inconsistent findings on the correlation between BMI and QoL among older adults have also been reported in previous studies. For example, some scholars found that BMI was not associated with QoL in older adults, especially in the Asian population (24, 25). However, other studies have reported that BMI was negatively associated with QoL in older adults (26–30). It is noteworthy that BMI was treated as both a continuous and categorical variable in previous research, which may have influenced the conclusions in these studies. Moreover, the relationship between BMI and QoL was found to be moderated by sex (28, 30), which suggests that the correlation between sex and QoL necessitates further investigation. Previous research has revealed inconsistent conclusions regarding the correlation between sex and QoL. For example, the results of a study with a large sample (*n* = 33,109), including older adults from China, Ghana, India, Russia, and South Africa, suggested that male older adults reported a better QoL than their female counterparts across all of the countries (31). A recent study conducted among Taiwanese Chinese older adults found that women experienced significantly higher QoL than men (32). However, previous findings concerning older adults in Brazil suggested that sex was significantly associated with QoL, but this relationship was moderated by other factors such as retirement status and physical or mental health status (33). Moreover, the results of a study on older adults in Iran suggested that sex was not associated with QoL of these older adults (34). Collectively, these inconsistent results indicate that further research needs to be conducted to explore the correlations of QoL with age, sex, and BMI.

Although some studies have revealed that QoL is associated with cognitive impairment in older adults, this relationship is complex (35, 36). Certain previous studies have shown that cognitive training intervention is protective against health-related QoL decline (37), which suggests that cognitive impairment may be a predictor of QoL. However, it has also been reported that poor QoL may result in low engagement in physical and social activities, which are risk factors for cognitive decline (38). Following the same logic, recent research has consistently demonstrated the predictive role of QoL in cognitive impairment and dementia onset in older adults (35, 39). A recent longitudinal study on middle-aged and older Chinese patients with diabetes, showed that participants’ health-related QoL (HRQoL) at Time 1 significantly predicted their cognitive impairment at Time 2 (40). Although previous mixed findings imply a bidirectional relationship between QoL and cognitive impairment, there is more evidence to support a predictive role of QoL on cognitive impairment.

Collectively, previous studies on the correlation of QoL with some of its predictors and consequences were mixed and we submit that as a result of this topic deserves further attention. However, it is worth noting that previous research has relied mainly on the variable-centered approach, which explores the associations among variables and provides an overall picture of the average relationships across the population (41). Variable-centered approaches are limited in that they overlook population diversity and intra-individual variability. In contrast, a person-centered approach identifies groups of individuals who share similar attributes or relations among attributes and thus allows researchers to examine how perceptions of QoL domains can be combined within individuals and relate to other variables (42). Latent profile analysis (LPA), as a person-centered approach, is based on a model and adopts strict statistical criteria to properly control class variables (43). As a result, it has been widely used in various settings. Although some studies explored the QoL profiles of specific (people with stoma, Islamic Inabah substance abusers, youth and young adults with sickle cell disease, homeless persons, long-term cancer survivors) (44–48), and general (Vietnamese people) (49) populations, research that has employed LPA to explore the QoL profiles of older adults and their relationships with antecedent or consequent variables is scarce. A recent study among Korean older adults explored HRQoL profiles and their relationships with selected predictors using a person-centered approach (latent cluster analysis [LCA]) (50). Four latent classes (stable type, physical disability type, emotional disability type and crisis type) emerged based on the HRQoL status measured using EQ-5D, which measures five aspects of HRQoL: mobility, self-care, usual activity, pain/discomfort, and anxiety/depression (51). Factors such as low income, household type, hypertension, number of falls, depression, and discomfort resulting from cognitive decline, happiness, frequency of contact, trust in the social environment, and participation in social activities significantly predicted latent classes. However, age and sex, which were considered potential predictors, were controlled. Moreover, only predictors, but not outcomes, of the HRQoL profiles were examined in this study.

To further advance our understanding of the QoL of older adults and its associations with antecedent and outcome variables, the current study aims to (1) explore the QoL profiles of a sample of community-dwelling older adults in HK using LPA; (2) examine the predictive role of age, sex, BMI, and depressive symptoms on the QoL profile membership; and (3) investigate profile differences in the distal outcome of cognitive impairment. Given the limited studies that have been conducted on these questions, no specific profile combinations were hypothesized. In relation to antecedent variables, no specific assumptions were made regarding age, sex, and BMI since mixed findings were reported in previous research. However, we hypothesized that older adults’ depressive symptoms would significantly predict QoL profile membership. It was also anticipated that the outcome variable of cognitive impairment would differ significantly across the QoL profiles.

## 2. Methods

### 2.1. Participants and procedure

A total number of 328 community-dwelling older adults from seven community centers for older adults in different districts of HK (Kowloon = 2, New Territories = 3, Hong Kong Island = 2) were invited to participate in this study. District community centers are a type of community support service at the district level that enable older adults to remain in the community while leading a healthy and dignified life. All people who are 60 years old qualify to register as members of community centers, join activities organized by the centers and seek assistance and support from the centers. This research was reviewed and approved by the Human and Animal Research Ethics Committee of a local university. The data were collected between March 2017 and April 2018 by eight research assistants who participated in a 3-h training workshop before data collection began. This training was carried out to homogenize and standardize the assessment methods in order to reduce inconsistencies among the data collected. Data collection was conducted at the district community centers using a one-on-one face-to-face interview format. The research assistants asked the participants questions according to the sequence prescribed by each assessment tool and recorded the responses. Written informed consent was obtained from each participant prior to data collection. After excluding incomplete and invalid responses, data from 259 older adults were identified as valid for the main analysis.

### 2.2. Measures

#### 2.2.1. Quality of life

The Chinese version of the World Health Organization Quality of Life-OLD (WHOQOL-OLD) (11) was used to measure the participant’s QoL. The initial WHOQOL-OLD module includes six facets (sensory abilities; autonomy; past, present, and future activities; social participation; death and dying; and intimacy) with four items for each facet. The responses were provided on a 5-point Likert scale ranging from 1 = “not at all” to 5 = “an extreme amount.” Three items from the sensory abilities facet (OLD_01, OLD_02, and OLD_10) and four items from the death and dying facet (OLD_06, OLD_07, OLD_08, and OLD_09) were reverse coded. Higher scores indicate a higher QOL. Facets and scale scores were transformed to a scale of 0–100. Previous research has demonstrated that the Chinese version of the WHOQOL-OLD has satisfactory validity and reliability (52). The WHOQOL-OLD demonstrated acceptable factorial validity, χ^2^ = 320.89, df = 237, CFI = 0.936, TLI = 0.926, RSEMA = 0.037 (90% CI: 0.026–0.047), SRMR = 0.065, and satisfactory internal consistency reliability (with composite reliability ranging from 0.65 to 0.857) in this study.

#### 2.2.2. Depressive symptoms

The Chinese version of the Geriatric Depression Scale-Short Form (GDS-SF) (53) was used to evaluate the participant’s depressive symptoms. The assessment tool includes 15 items with each item being rated on a dichotomous scale of “yes” or “no” yielding a score range of between 0 and 15. A higher score indicates a higher level of depressive symptoms. Participants with a score equal to or greater than eight were considered likely to be depressed. Previous research has consistently demonstrated that the Chinese version of the GDS-SF has satisfactory validity and reliability (53–55).

#### 2.2.3. Cognitive impairment

The Cantonese version of the Mini-Mental State Examination (MMSE) (56) was used to assess the cognitive impairment of participants. The Cantonese version of the MMSE (C-MMSE) is an 11-item instrument. Most items of the MMSE were directly translated and used in the C-MMSE during this study, but major adaptations had to be made for several items. For example, the phrase “no if’s, and’s or but’s” was replaced with the phrase “姨丈買魚腸” which is translated to “Uncle buys fish intestine,” an alliteration in Cantonese. The maximum score for the C-MMSE is 30, and a cut-off value of 19/20 is recommended as an indication that cognitive impairment may require further evaluation. Previous research has demonstrated that the C-MMSE displays satisfactory validity and reliability among Chinese older adults in Hong Kong (56).

### 2.3. Plan of analyses

Descriptive statistics and correlations of all main variables were calculated using the IBM Statistical Package for Social Science (SPSS) (Version 22 IBM, Armonk, NY). LPA using the six QoL domains of the WHOQOL-OLD as profile variables was conducted using Mplus (Version 7.31) with Robust maximum likelihood (MLR) to explore solutions from one to four profiles. A combination of relative fit indices, including the log likelihood value (LL), Akaike Information Criterion (AIC), Bayesian Information Criterion (BIC), Adjusted Bayesian Information Criterion (ABIC), entropy, Lo–Mendell–Rubin adjusted Likelihood Ratio test (aLRM), and bootstrapped likelihood-ratio test (BLRT), were employed to choose the best solution. Smaller values of the AIC, BIC, and ABIC and higher values of the LL and entropy are indicative of a better solution. The aLMR compares the estimated model (k) with a model that has one class less than the estimated model (k-1). Non-significant *p* values support the k-1 profile model. Higher entropy suggests a more accurate classification, ranging from 0 to 1. A multivariate analysis of variance (MANOVA) was conducted with the QoL profiles as the independent variable and six QoL domains as the dependent variables to examine the criterion validity of the LPA. Multinomial logistic regression using the R3STEP function (57) in Mplus was conducted to examine whether profile membership could be predicted by antecedent variables including age, sex, BMI, and depressive symptoms. Age and BMI were treated as continuous variables, and sex and depressive symptoms were dummy-coded (woman = 0, man = 1; GDS score lower than 8 = 0, GDS score equal to or above 8 = 1). The Bolck-Croon-Hagenaars approach (BCH) (58) approach in Mplus was employed to examine how the distal outcome of cognitive impairment differs across QoL profiles.

## 3. Results

### 3.1. Descriptive statistics and correlations

The descriptive statistics of the variables are presented in Table 1. Participants reported extremely low scores on the death and dying domains and moderate-to-high scores on the other five QoL domains. There were no significant sex differences in any of the variables except for height, body weight, and BMI. The bivariate correlations among the variables are presented in Table 2. Age and BMI were found to be significantly associated with certain QoL domains (autonomy and intimacy), cognitive impairment, and depressive symptoms. Furthermore, the autonomy domain was associated with depressive symptoms and cognitive impairment, while the death and dying domain was associated with depressive symptoms. In addition, low-to-moderate correlations were found among the QoL domains.

**Table 1:** Descriptive statistics of variables.

Variables	Total (*n* = 259)	Male (*n* = 57)	Female (*n* = 202)	*t*
Age	78.46 (7.79)	78.37 (8.00)	78.48 (7.56)	−0.95
Height	151.96 (7.18)	159.05 (6.13)	149.95 (6.11)	9.926^***^
Weight	56.17 (9.05)	59.04 (9.71)	55.36 (8.71)	2.748^**^
BMI	24.34 (3.50)	23.40 (3.55)	24.60 (3.45)	−2.301^*^
SAB	55.12 (22.80)	59.98 (21.52)	53.74 (23.01)	1.832
AUT	70.10 (18.88)	70.50 (19.15)	69.99 (18.85)	0.182
DAD	20.51 (22.85)	15.39 (18.52)	21.95 (23.78)	−1.924
PPF	65.08 (20.91)	64.91 (22.15)	65.13 (20.61)	−0.069
SOP	76.13 (18.14)	73.14 (19.12)	76.98 (17.82)	−1.415
INT	62.11 (26.18)	61.80 (24.37)	61.35 (26.68)	0.878
GDS	7.15 (2.08)	7.10 (2.32)	7.17 (2.01)	−0.201
MMSE	23.01 (5.43)	23.91 (5.56)	22.75 (5.37)	1.427

***p < 0.001; **p < 0.01; *p < 0.05; BMI, body mass index; SAB, sensory ability; AUT, autonomy; DAD, death and dying; PPF, past, present and future activities; SOP, social participation; INT, intimacy; GDS, geriatric depression scale; MMSE, mini-mental state examination.

**Table 2:** Bivariate correlations among variables.

	Age	Height	Weight	BMI	SAB	AUT	DAD	PPF	SOP	INT	GDS
Height	−0.298^**^										
Weight	−0.174^**^	0.441^**^									
BMI	0.007	−0.162^**^	0.809^**^								
SAB	0.051	−0.006	−0.042	−0.036							
AUT	−0.142^*^	0.067	0.084	0.061	0.020						
DAD	−0.113	−0.015	−0.014	−0.008	−0.135^*^	−0.064					
PPF	−0.119	0.043	−0.041	−0.059	−0.004	0.391^**^	0.101				
SOP	−0.042	−0.059	−0.073	−0.029	0.073	0.374^**^	−0.040	0.594^**^			
INT	−0.147^*^	0.082	−0.078	−0.127^*^	0.103	0.325^**^	0.032	0.510^**^	0.469^**^		
GDS	−0.450^**^	0.274^**^	0.078	−0.100	−0.079	0.208^**^	0.096	0.016	0.019	0.092	
MMSE	−0.126^*^	−0.040	−0.044	−0.025	−0.122	−0.159^*^	0.161^**^	−0.054	−0.086	−0.081	−0.062

**p < 0.01; *p < 0.05; BMI, body mass index; SAB, sensory ability; AUT, autonomy; DAD, death and dying; PPF, past, present and future activities; SO, social participation; INT, intimacy; GDS, geriatric depression scale; MMSE, mini-mental state examination.

### 3.2. Latent profile analysis and profile description

**Figure 1:**
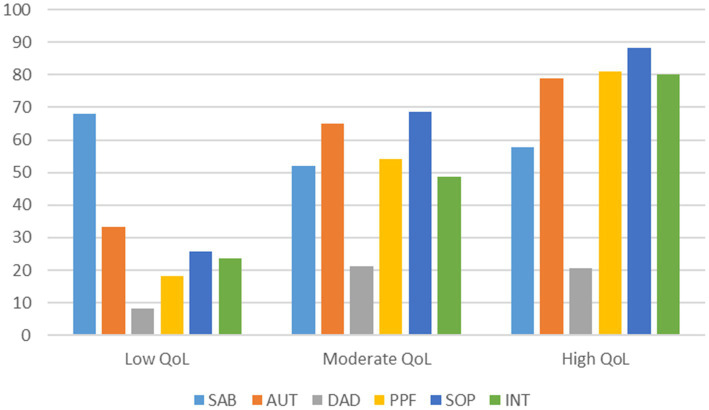
Three QoL profiles emerged from latent profile analysis. SAB, sensory ability; AUT, autonomy; DAD, death and dying; PPF, past, present and future activities; SOP, social participation; INT, intimacy.

Table 3 presents the statistical indicators and tests for the LPA models. We started with a solution with one profile as the minimum and then extended it progressively with more profiles until the addition of one more profile did not significantly improve the model fit. The values of AIC, BIC, and SABIC decreased as the number of profiles increased. The aLMR in the four-profile model was nonsignificant, which indicated that a solution with three profiles was superior to the two-profile model. Moreover, it was found that the entropy value reached a peak in the model with three profiles. Therefore, a solution with three profiles was considered to be a satisfactory model. Figure 1 illustrates the three profiles. Profile 1 (*n* = 9; 3.5% of the sample) is characterized by low scores on five out of six QoL domains (with the exception of the sensory ability domain) and is labeled as a Low QoL profile. Profile 2 (*n* = 132; 50.9% of the sample) was characterized by moderate scores on five out of six QoL domains (with the exception of the death and dying domain) and labeled as a Moderate QoL profile. Profile 3 (*n* = 118; 45.6% of the sample) is characterized by high scores on four out of six QoL domains (with the exception of the sensory ability and death and dying domains) and is labeled as a High QoL profile.

**Table 3:** Latent profile fit statistics for models based on the six quality of life domains.

Model	LL	Scaling	#FP	AIC	BIC	SABIC	Entropy	aLMR *p*-value	BLRT *p*-value	Smallest class %
1-Profile	−6966.989	1.071	12	13957.979	14000.661	13962.616	–	–-	–	
2-Profile	−6873.533	1.221	19	13785.066	13852.646	13792.409	0.710	0.0062	<0.001	42.857
3-Profile	−6817.770	1.023	26	13687.541	13780.018	13697.589	0.820	<0.001	<0.001	3.475
4-Profile	−6808.636	1.186	33	13683.271	13800.646	13696.025	0.723	0.7015	0.1463	3.475

LL, model log-likelihood; Scaling, scaling factor associated with MLR log-likelihood estimates; #FP, number of free parameters; AIC, Akaike information criteria; BIC, Bayesian information criteria; SABIC, sample-size adjusted BIC; aLMR, adjusted Lo–Mendell–Rubin likelihood ratio test; BLRT, bootstrap likelihood ratio.

The results of the MANOVA revealed a significant multivariate effect for profiles on QoL domains, Pillai’s trace = 0.946, *F* (12, 504) = 32.675, *p* < 0.001, *η*^2^ = 0.438. Follow-up univariate F-tests suggested significant effects of profiles on four out of six QoL domains (with the exception of the sensory ability and death and dying domains). All statistics are presented in Table 4. These results support the interpretability of LPA results.

**Table 4:** Group differences on profile variables.

	Profile 1 (*n* = 9)		Profile 2 (*n* = 132)		Profile 3 (*n* = 118)		F	*p*	*η^2^*	Profile difference
	M	SD	M	SD	M	SD	(2, 256)			
1. SAB	68.06_a_	18.07	51.89_a_	21.12	57.73_a_	24.35	3.618	0.028	0.027	1 = 2 = 3
2. AUT	33.33_a_	18.75	64.87_b_	17.17	78.76_c_	14.87	46.834	<0.001	0.268	3 > 2 > 1
3. DAD	8.25_a_	10.55	21.32_a_	23.83	20.54_a_	22.36	1.383	0.253	0.011	1 = 2 = 3
4. PPF	18.06_a_	10.10	54.12_b_	14.17	80.93_c_	13.17	176.524	<0.001	0.580	3 > 2 > 1
5. SOP	25.69_a_	16.07	68.61_b_	12.74	88.40_c_	10.73	167.353	<0.001	0.567	3 > 2 > 1
6. INT	23.61_a_	19.71	48.77_b_	22.54	79.98_c_	17.13	93.205	<0.001	0.421	3 > 2 > 1

Bonferroni correction was adopted for multiple group comparisons with *p* values were significant at 0.017. SAB, sensory ability; AUT, autonomy; DAD, death and dying; PPF, past, present and future activities; SOP, social participation; INT, intimacy. Values shared same subscript mean non-significant differences on values across groups.

### 3.3. Antecedent and consequent variables of QoL profiles

Table 5 presents the results of the multinomial logistic regression. It was found that depression, but not age, sex, or BMI, was significantly associated with profile membership, which suggests that the age, sex, and BMI of older adults may not contribute to their QoL status. However, older adults with depression were more likely to be members of the Low QoL profile relative to the Moderate QoL profile (OR = 5.512, *p* < 0.01) and High QoL profile (7.156, p < 0.01). In other words, older adults with depression were five and six times as likely to be allocated to the Low QoL profile relative to the Moderate and High QoL profiles as were their non-depressed counterparts, respectively. Table 6 presents the results of the difference tests on cognitive impairment across profiles using the BCH procedure. There were no significant differences found in cognitive impairment across the three QoL profiles (*χ*^2^ = 3.955, *p* = 0.138).

**Table 5:** Results of multinomial logistic regressions for the effects of predictors on profile membership.

	Profile 1 vs. 3^a^		Profile 2 vs. 3^a^		Profile 1 vs. 2^a^	
	Coef. (SE)	OR	Coef.	OR	Coef.	OR
Age	0.016 (0.036)	1.016	0.035 (0.021)	1.035	−0.019 (0.036)	0.981
Gender	−0.982 (0.762)	0.374	0.160 (0.376)	1.173	−1.141 (0.771)	0.319
BMI	−0.016 (0.092)	0.984	0.059 (0.046)	1.061	−0.075 (0.092)	0.927
GDS	1.968^**^ (0.895)	7.156	0.261 (0.322)	1.298	1.707^**^ (0.899)	5.512

Dummy coding (GDS score lower than 8 = 0; GDS score equal to or above 8 = 1; Female = 0; Male = 1); Profile 1 = Low QoL; Profile 2 = Moderate QoL; Profile 3 = High QoL; SE, standard error of the coefficient (Coef); OR, odds ratio.

a Reference group.

*p < 0.05, **p < 0.01.

**Table 6:** BCH results for the differences on the cognitive impairment across latent profiles.

	Profile 1	Profile 2	Profile 3	Comparison
MMSE	22.30	22.509	23.609	1 = 2 = 3

MMSE, mini-mental state examination.

## 4. Discussion

Previous research on the QoL of older adults mainly employed variable-centered approaches to explore the average relationships of QoL with related antecedent and outcome variables. Adding to the body of knowledge in this subject area, the results of our study employed a person-centered approach to explore whether subgroups with distinct QoL profiles based on QoL domains can be identified among a sample of community-dwelling older adults in HK. Furthermore, we explored the associations of predictors (age, sex, BMI, and depression) with and differences in distal outcome (cognitive impairment) across QoL profiles. This study contributes to the literature in the field by increasing what is known about QoL profiles of older adults and examining the relationships between antecedents, QoL profiles, and the consequences from a person-centered perspective.

### 4.1. Quality of life profiles

To our knowledge, this is the first study to investigate this particular topic using latent profile analysis among older adults in HK. Three QoL profiles, namely Low QoL, Moderate QoL and High QoL, emerged from the analysis, and significant differences in most of the profile variables across the three QoL profiles were observed. Older adults with a Low QoL profile displayed a similar pattern, with low scores on five out of six QoL domains and an above-average score on the sensory ability domain. Accordingly, the sensory functioning of older adults in this group was good, but they experienced limited autonomy, were dissatisfied with their lives, appeared to be stuck in the past, did not participate frequently in social activities, lacked intimate relationships, and had negative feelings and thoughts about death or dying. This profile is similar to the “Low QoL” class some older adults in Sri Lanka fall into after utilization of K-mean cluster analysis, in which participants reported low scores on physical, mental, social, functional, environmental, and spiritual domains (59). It also shares similar characteristics with the profile of “crisis type” that emerged from Korean older adults using latent class analysis, in which older adults reported low scores on mobility, self-care, usual activities, pain/discomfort (50). Older adults with a High QoL profile reported a similar pattern of high scores on four out of six QoL domains, a moderate score on the sensory ability domain, and a low score, although slightly higher than their counterparts in the Low QoL profile in the death and dying domain. This means that although the participants might have had some sensory functioning concerns, they could make their own decisions, participate in regular social activities, have strong intimate relationships with others, and are satisfied with their lives in general. Surprisingly, they also reported low scores on the death and dying domain, similar to their counterparts in both Low QoL and Moderate QoL profiles. As illustrated in Table 1, the mean score of the sample in the death and dying domain was low. One possible reason may be that Chinese older adults in HK are sensitive to the topic of death and dying and overreacted to questions related to the topic. Unfortunately, there is no data available on Chinese older adults in HK on this subject from other studies. However, existing data on Chinese older adults in Mainland China using the same measure (WHOQOL-OLD) revealed a moderate to high score in this domain. Further research may need to be conducted on this issue in the future. The High QoL profile that emerged in this study shared similar characteristics with the “stable type” profile observed among Korean older adults (50) and “High QoL” profile of older adults in Sri Lanka (59), in which participants reported high scores on all QoL domains. However, given that different measures were used across the three studies, the profiles shared similar patterns of scoring in some related domains. Regarding the Moderate QoL profile, the participants in this profile reported a similar pattern of moderate scores on five out of six QoL domains but a low and similar score with their counterparts in the High QoL profile in the death and dying domain. For this particular profile, it may be understandable, but no comparable profiles have emerged in respect of Korean older adults. The other two profiles that emerged from Korean older adults were the “physical disability type” and “emotional disability type.” The former is characterized by extremely low scores on the mobility and pain/discomfort domains, whereas the latter is characterized by extremely low scores on the anxiety/depression domain (50). It is noteworthy that more than half of the participants (50.9%) were allocated to the Moderate QoL profile in the current study.

### 4.2. Antecedent and consequent variables of QoL profiles

Our results suggest that depression, but not age, sex, or BMI, significantly predicted QoL profile membership. The significant correlation between depression and QoL is consistent with previous research findings and provides further support for this relationship from a person-centered approach. In our analysis, depressive symptoms were dummy-coded (0 or 1) using a cut-off value of 8 for the GDS score, with a score lower than 8 coded as non-depression (0) and scores equal to or higher than 8 coded as depression (1). It was found that older adults with depression were more likely to be allocated to the Low QoL profile than to the Moderate or High QoL profiles. This result reasonably explains why older adults in the Low QoL profile had moderate scores in the sensory ability domain, but low scores in other domains. A moderate score on the sensory ability domain indicated that the sensory functioning of these older adults was above average and did not negatively influence their daily lives. Therefore, their low scores on autonomy, social activity participation, attitude toward the past, present, and future, death and dying, and intimacy with others may be the result of their depression since depressed individuals were more likely to isolate themselves from others, to be stuck in the past, and to be pessimistic about the present and future. Our results, together with previous findings, suggest that the prevention and treatment of depression is an important strategy to be pursued in improving and maintaining QoL in older adults (9, 19). An insignificant relationship between age and QoL was observed in this study, which is consistent with previous findings in Chinese older adults (21) as well as in other older adult populations (22). Practitioners, such as social workers, and family members of older adults are encouraged to abandon the stereotype that aging will inevitably result in poor QoL and rather utilize these findings to strengthen older adults’ beliefs that they are in control of their QoL. The nonsignificant relationship found between BMI and QoL in this study is consistent with previous findings in the Asian population (24, 25). It should however be noted that BMI was treated as a continuous variable in our analysis, but that this is unlike in previous studies that treated BMI as a categorical variable (30). However, in Lee’s study, where BMI was treated as a categorical variable during analysis, the results revealed an insignificant relationship between BMI and QoL (24). Further research is required to investigate the relationship between BMI and QoL since the relationship may not be linear (30) and a more complex relationship may be observed among older adults with different weight statuses. Sex was not found to significantly predict QoL profile membership in this study, which is consistent with findings of a study on older adults in Iran (34), but differs from findings of further studies on older adults in other countries (31–33). Previous research has demonstrated that sex played a role in predicting QoL of older adults, with either men (31) or women (32) being significantly associated with higher QoL. It must be noted that the results of this study should be interpreted with caution because the number of men in the sample was relatively small. We submit that further research should be conducted in the future to explore this relationship by attempting to recruit more men to participate in the prospective studies.

Surprisingly, we did not observe any significant difference in the levels of cognitive impairment across the QoL profile, although a decrease was evident in participants of the Low QoL profile (reporting the lowest scores), while participants in the High QoL profile reported the highest scores. Although this result is inconsistent with previous findings, it is important to note that this is the first study to employ a person-centered approach to explore the association between QoL profile and cognitive impairment. We submit that further research should be conducted to explore the relationship between QoL and cognition using the same approach in a larger sample.

### 4.3. Limitations and future direction

To the best of our knowledge, this is the first study to investigate QoL profiles and their associations with predictors and outcomes using a person-centered approach among a sample of community-dwelling older adults in HK. Although this study contributes to the literature in the field of QoL research by providing new evidence using a person-centered approach, some limitations should be noted. First, the sample size was relatively small, although it was sufficient for statistical data analysis. Researchers are encouraged to investigate this research topic in the future using a larger sample, focusing on the need to recruit more men as participants. Second, data was collected in district community centers, this means we could only collect data from older adults who were willing to visit these centers regularly. In other words, the older adults who participated in this study may not best represent all older adults in Hong Kong and scholars should take care when trying to generalize our results. We submit that future researchers should consider alternative methods to recruit participants, such as cold calling and home visits in order obtain a more diverse sample. Third, relationships between socioeconomic and social vulnerability factors (e.g., education attainment, marital status, and individual incomes) and QoL of older adults were not examined in this study, which is one of the limitations of the current study. Future researchers are encouraged to shed light on these research questions using person-centered approach, which will inform policy makings and practical works by providing tailor-made interventions or strategies in the future. Finally, this study has a cross-sectional design; thus, causal relationships among variables cannot be made. Future research should employ a longitudinal design using latent transition analysis to investigate changes in the latent profile of QoL over time and explore their longitudinal relationship with predictors and outcomes.

## 5. Conclusion

The findings of the current study add to the growing body of literature exploring QoL profiles and their associations with antecedent and outcome variables. The results of our study improve the extent and quality of what is known on the subject, supplementing the results of previous studies by utilizing a person-centered approach to examine the QoL profiles of community-dwelling older adults. Three distinctive QoL profiles emerged, and depression, but not sex, age, or BMI, was found to significantly predict profile membership. No significant differences in cognitive impairment were observed across the three profiles.

## Data availability statement

The raw data supporting the conclusions of this article will be made available by the authors, without undue reservation.

## Ethics statement

The studies involving human participants were reviewed and approved by Hong Kong Baptist University. The participants provided their written informed consent to participate in this study.

## Author contributions

J-DL: conceptualization and resources. GL, DW, YZ, and J-DL: methodology and formal analysis. WL, DW, XW, and YZ: validation. J-DL and YZ: investigation. GL, WL, YZ, and XW: data curation. GL and WL: writing–original draft preparation. J-DL and DW: writing–review and editing and supervision. All authors meet the criteria for authorship according to their contributions to the manuscript. All authors contributed to the article and approved the submitted version.

## Funding

This research was supported by Sanming Project of Medicine in Shenzhen, China (Grant number: SZSM201812052).

## Conflict of interest

The authors declare that the research was conducted in the absence of any commercial or financial relationships that could be construed as a potential conflict of interest.

## Publisher’s note

All claims expressed in this article are solely those of the authors and do not necessarily represent those of their affiliated organizations, or those of the publisher, the editors and the reviewers. Any product that may be evaluated in this article, or claim that may be made by its manufacturer, is not guaranteed or endorsed by the publisher.
